# Four decades of overdose prevention centres: lessons for the future from a realist review

**DOI:** 10.1186/s12954-025-01178-z

**Published:** 2025-03-20

**Authors:** Jolie R. Keemink, Alex Stevens, Sam Shirley-Beavan, Zarnie Khadjesari, Gillian W. Shorter

**Affiliations:** 1 University of Kent, Canterbury, UK; 2 University of Sheffield, Sheffield, UK; 3 University of East Anglia, Norwich, UK; 4 Queen’s University Belfast, Belfast, UK

**Keywords:** Harm reduction, Realist review, Overdose prevention centre, Drug consumption room, History

## Abstract

**Background:**

Overdose prevention centres (OPCs) are spaces where people can consume previously obtained illicit drugs under the supervision of staff who can intervene to prevent and manage overdose. They have been provided in Europe and elsewhere for nearly 40 years, initially in response to the epidemic of HIV/AIDS. We can learn from their operation history to inform future developments in harm reduction services.

**Methods:**

We carried out a realist review of 391 documents, reported according to the RAMESES I guidelines, and carried out realist synthesis of these documents.

**Results:**

We present a full realist programme theory of OPCs, with a diagrammatic logic model, of how the contexts and mechanisms of OPCs combine to produce various outcomes for service users and their communities in different settings. Three specific causal pathways were evidenced through which OPCs produce their outcomes for particular groups in specific contexts of housing status, gender identity and ethnicity, and local drug markets, with frequency of use, legal and political contexts, and stigma as overlapping contextual factors. Key OPC interventions include the provision of a safe and hygienic consumption space, safe consumption education, timely overdose response, and protection from drug scene and gender-based violence. These can trigger the underlying mechanisms of safety, trust, social inclusion, engagement, autonomy, and empowerment when supported with health care and other services, including detoxification and opioid agonist treatment. The combinations of these contexts and mechanisms create important outcomes for individual service users, for the communities they live in, and for wider society. We also describe causal pathways that can lead to unintended, adverse outcomes.

**Conclusion:**

This review provides useful information for policy makers, practitioners, and researchers on how to implement and evaluate OPCs in future to maximise their benefits; an important task in the context of the ongoing public health crises of drug poisoning deaths in North America and the UK, and the possibility of increasing deaths from synthetic opioids in Europe and elsewhere.

**Supplementary Information:**

The online version contains supplementary material available at 10.1186/s12954-025-01178-z.

## Introduction

To reduce harms related to street-based consumption of illicit drugs, several European countries have added overdose prevention centres to the range of harm reduction services that they provide [1]. These are non-residential spaces where people can consume illicit drugs that they have obtained elsewhere, in the presence of staff who can intervene to prevent and manage any overdoses that occur.^1^ Their history now goes back nearly 40 years to the mid-1980s. The first officially sanctioned OPC was established in Switzerland in 1986 [3]. By 2004, there were 13 OPCs operating in seven Swiss cities [4]. OPCs have since been established in countries across Europe, North America, Latin America, and Australia. Originally, such services were introduced to provide safe spaces and reduce the transmission of HIV and viral hepatitis between people who inject drugs [4–6]. Their role in reducing overdoses became more important following the substantial rise in several countries, including Canada, the USA and UK [7–9]. There is a large body of research on the effectiveness of OPCs. The previously reviewed evidence suggests that OPCs are cost-effective, reduce overdose-related mortality, as well as HIV and HCV infections, reduce the number of ambulance call outs, decrease public nuisance, disorder, and drug-related litter, and support people who use drugs to enter treatment [10–17]. Qualitative studies in this field show that people who use drugs support the use of OPCs; they also show these places offer refuge from street violence and adverse police contact, and a safer place to consume drugs leading to a reduction in injection-related risks [18–23]. They can be a site for the provision of other services, including the checking of the contents of drugs that people intend to use [24, 25]. OPCs cannot, on their own, resolve all the problems related to ongoing social exclusion of people who use drugs, and an unregulated drug market. They may form part of a comprehensive response to these problems.

Despite the relatively large number of studies on the effectiveness of OPCs, there is a lack of knowledge on the underlying causal mechanisms which produce their effects. To inform the future development of these services, we need to examine what works, for whom, in what context, and why. We have already written about the main causal pathway through which OPCs produce their outcomes by providing a feeling of safety, trust, and social inclusion [26], but there is more to learn from a full programme theory of OPCs. This involves building an initial programme theory of how a complex intervention is thought to work, and subsequently examines empirical data to confirm, contradict, or change the programme theory [27]. Realist analysis provides explanations in the form of contingent combinations of contexts, mechanisms, outcomes (CMO) [28]. We can also learn from OPCs that did not produce the intended outcomes, in Europe and elsewhere.

This article presents data and analysis from the first realist review of the literature on past and presently operating OPCs. In order to inform the future development of harm reduction services. Through analysis of 391 documents, we develop an overall programme theory and three more specific causal pathways within this overall theory. We also provide a ‘dark logic’ model of unsuccessful CMOs. It is especially important for policy makers and harm reduction providers in Europe to learn from the experience of North America, where changes in illicit^2^ opioid supply have led to a devastating crisis of opioid poisonings [8, 9]. The future for people who use drugs in Europe is also threatened by the arrival of powerful synthetic opioids [29] and this sits alongside an ongoing European priority to address drug-related deaths, e.g. in the UK [7]. It is a matter of urgency to learn from the four decades of experience with OPCs about how to save lives and promote health and wellbeing though inclusion of OPCs in the future development of a comprehensive response to the social exclusion and health needs of people who use drugs.

## Method

### Design

This review was carried out following the RAMESES I guidelines [30]. A protocol for the review was registered on PROSPERO (CRD42023414273).

This review aimed to answer the following two research questions:

1. What are the main contexts, mechanisms, and outcomes of OPCs?
2. Regarding OPCs, what works, for whom, in what circumstances?

Our initial programme theory of OPCs (see Appendix 1) was informed by previous reviews of the literature [9–16], and refined in consultation with key stakeholders, including people who use drugs, academics, practitioners in drug treatment and harm reduction services, and advocacy experts, across Europe and internationally. The group included members of the European Network of People who Use Drugs, who also discussed our initial theory and findings with peers in their network. We also presented the initial programme theory and initial findings to meetings of the Drug Science Enhanced Harm Reduction Working Group; a network of British practitioners and academics who regularly share information about developments in harm reduction. This group also includes people with direct experience of injecting drug use.

### Inclusion criteria

Realist reviews are inclusive of a wide range of research types. There were no limitations to the type of research design we included. Documents were included if they could provide relevant, rich, and rigorous empirical data [31]. We excluded feasibility studies, opinion pieces, commentaries, and policy reports that did not include empirical data on operating OPCs. There was no start date to the literature search; the first document found in our searches was published in 1999 [32]. Due to limited funding, we excluded documents that were not in English. This also helped us gather insights from older reports that were not in English. For example, Zobel and Duboid-Arber’s appraisal of evidence on Swiss OPC’s contained information from earlier research published in both German and French [4].

### Search strategy

We scrutinised search strategies employed in previous reviews and developed these by making our search strategy more comprehensive. Our search terms were:

“overdose prevention cent*” OR “overdose prevention site*” OR “overdose prevention program*” OR “overdose prevention facilit*” OR “supervised inject* service*” OR “supervised inject* facilit*” OR “supervised inject* centre*” OR “supervised inject*” OR “supervised inject* program*” OR “supervised inject* room*” OR “supervised fixing room*” OR “supervised drug consumption facilit*” OR “supervised injectable maintenance clinic*” OR “safe* inject* facilit*” OR “safe* inject* space*” OR “safe* consumption space*” OR “drug consumption room*” OR “drug consumption facilit*” OR “medically supervised inject* cent*” OR “fix* room*” OR “safe* environment intervention*” OR “shooting galler*”

**Fig. 1:**
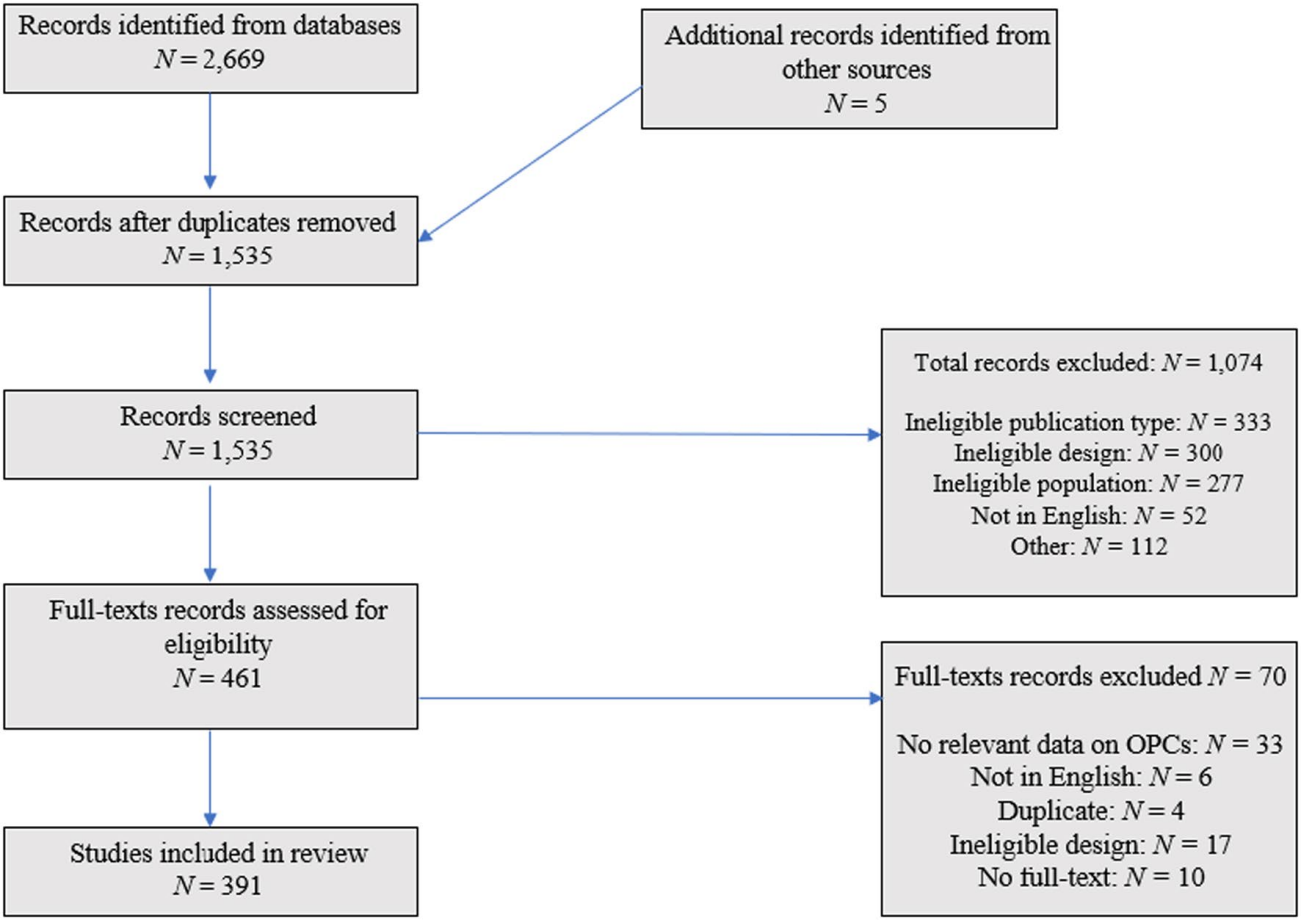
Flowchart of the review screening process for the realist review of OPCs

The following bibliographic databases were searched for relevant articles between 18 and 20 April 2023: PubMed, Scopus, and Web of Science. We also searched for grey literature in the online bibliography maintained by the International Society for the Study of Drug Policy. The database of documents from the recent Drug Science Rapid Review of OPC Evidence [17] was checked for missed references. Table 1 presents the number of identified documents for each database. We consulted with stakeholders to identify further eligible documents, and used ResearchRabbit to search for relevant documents that we may have missed (no additions were found). In Rayyan, we excluded duplicates and screened the abstracts of the 1,536 identified documents. Ten percent of abstracts were double screened to ensure a consistent approach between screeners. Conflicts were resolved by discussion between the screeners (JK and AS). This was an iterative process which and ongoing process that did not lend itself to the calculation of a precise score for inter-rater reliability. The full texts of selected documents (N = 461) were imported into Zotero. Of these, 70 were found—during the process of extracting and coding data—not to fit the inclusion criteria. This left 391 documents which met our inclusion criteria and so are covered by this review. See Fig. 1 for a flow chart of the screening and selection process.

**Table 1:** Summary of the searches on four databases and the found results for the realist review on OPCs

Search engine	Hits	Search date
SCOPUS	1008	18/04/23
Pubmed	664	18/04/23
Web of science	986	18/04/23
International society for the study of drug policy	10	20/04/23

We reviewed 132 documents that were published in English that provided information on OPCs that operated in non-Anglophone countries.

### Data extraction and analysis

The 391 documents saved in Zotero were uploaded to NVivo for data extraction and thematic analysis following similar methods to those outlined by Wiltshire and Ronkainen [33]. Three researchers shared the coding of the documents and highlighted relevant sections of the documents for extraction (AS, JK, SSB). Our initial programme theory formed the foundation for our provisional coding structure. It included expected and possible contexts, mechanisms and outcomes of OPCs (see Appendix 1). We coded abductively, and researchers added and changed codes as the analysis developed [34]. Researchers met weekly to discuss progress and new codes. The final coding framework comprised 322 codes.

The final step of our analysis was retroduction. This is a form of inference that derives provisional conclusions about generative structures underlying the empirical data [34]. Retroduction examines how the outcomes of an intervention are caused. It also includes counter-factual reasoning, considering what would have happened in the absence of identified contexts and mechanisms. It is in retroduction that we identified the causal pathways by which interventions lead to outcomes. This final phase of analysis also involved consultation with our stakeholder group, including people who use drugs.

### Ethics

Since this project is concerned with secondary data analysis, ethical approval was not required.

## Results

### Overview of study characteristics

**Fig. 2:**
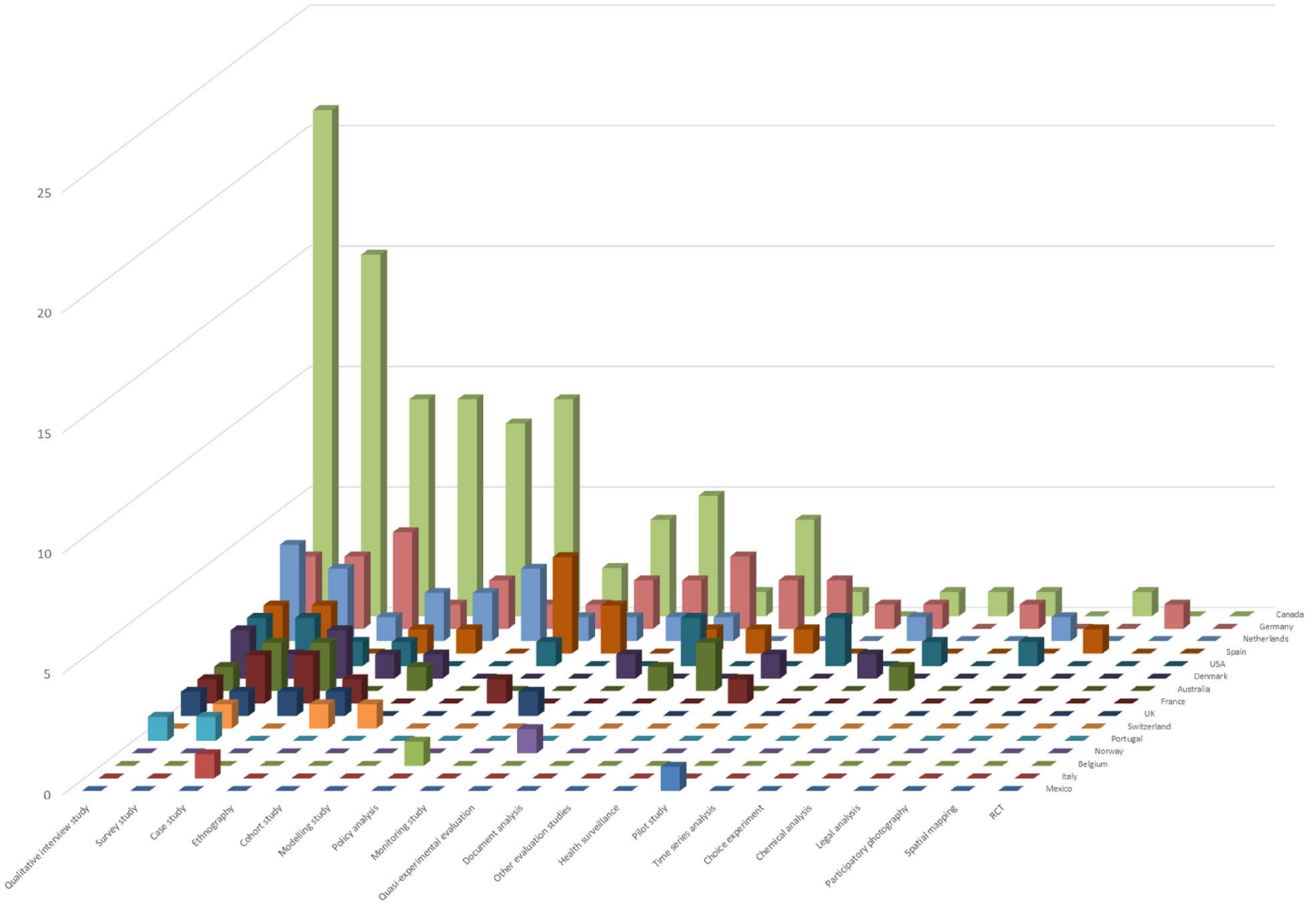
Number of primary studies on OPCs included in the review, by country and research method of the study

A total of 391 documents were included in the final review. OPCs in 17 different countries were reported in these documents, with most documents covering OPCs in Canada (*N* = 201), Australia (*N* = 65), and Germany (*N* = 62). We identified 89 different individual OPCs that were included in the selected documents. This compares to reports of over 100 OPCs being in operation worldwide in 2023. Many documents included data from INSITE, in Vancouver, Canada (*N* = 74*).* Just as many individual OPCs in Germany were mentioned in selected documents as in Canada (30 in each country), but there were far fewer primary research studies on European OPCs. Qualitative studies, surveys and case studies were the most common research designs internationally, including in Europe as shown in Fig. 2.

We found no randomised controlled trials of OPCs. As has previously been noted, it would be very difficult to run such experimental studies on OPCs, but their absence limits the confidence of some researchers and commentators in the findings of previous research on OPC outcomes [35, 36]. The documents we selected also referred to OPCs existing in Greece and Luxembourg. More recently, OPCs have opened in Colombia and Iceland [37]. Our search did not find primary studies of OPCs in these countries.

### Contexts, mechanisms and outcomes in a programme theory of OPCs

In the section below, we describe three key context-mechanism-outcome configurations identified in our analysis of the extracted data. These are defined as follows:

- A **context** is a characteristic of the environment or persons involved in an intervention which affects its mechanisms and outcomes. Contexts can pre-exist the delivery of the intervention, or be dynamically affected by the intervention in operation.
- A **mechanism** is a causal process which is not directly observable that is triggered by observed OPC intervention components which combine with the contexts to produce outcomes.
- An **outcome** is a change that occurs that is caused by the mechanisms, as affected by the contexts.

**Fig. 3:**
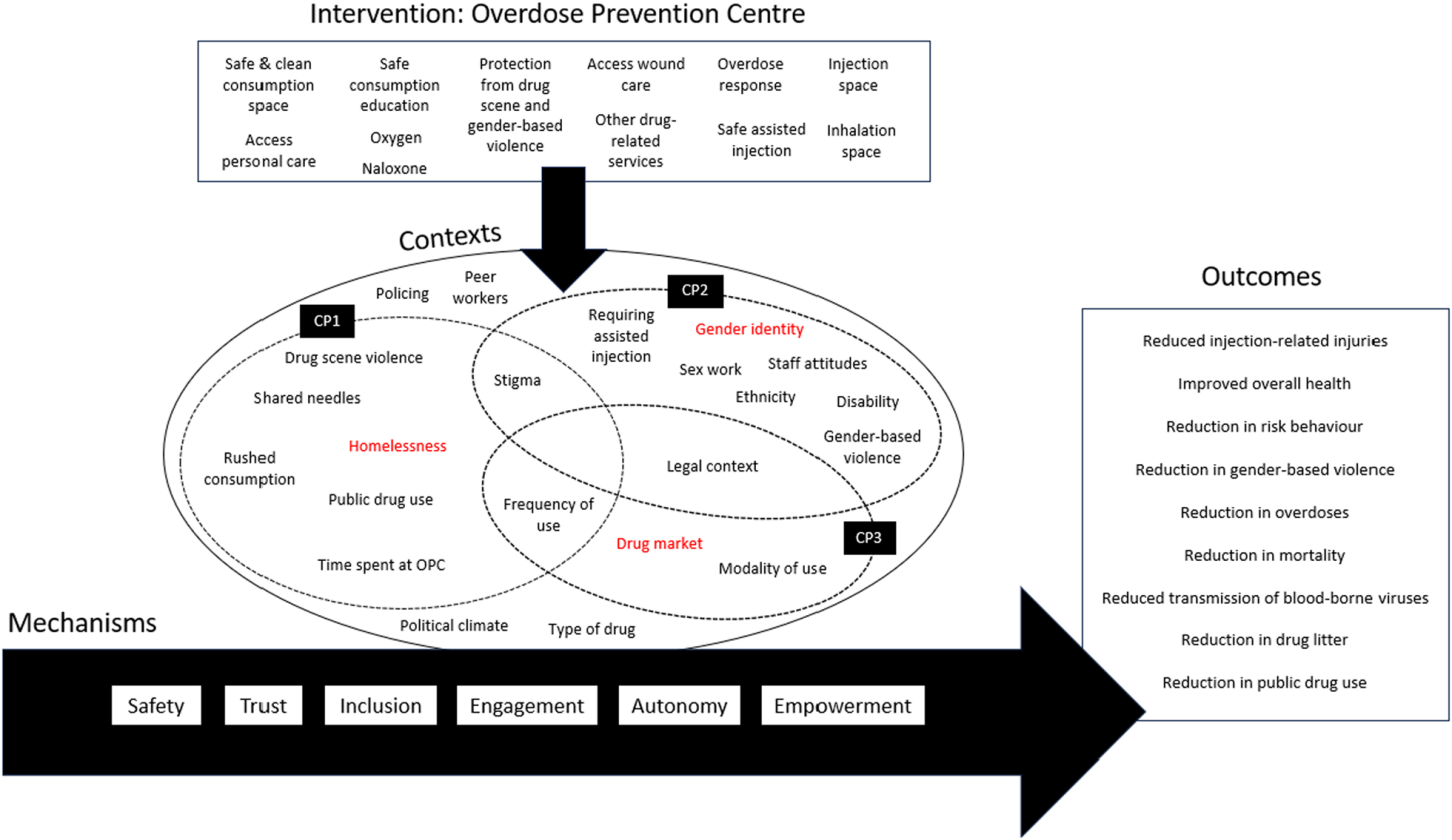
A Programme theory of overdose prevention centres. *Note*: CP1 is the causal pathway considering homelessness as a key contextual factor. CP2 is the causal pathway considering gender identity as a key contextual factor. CP3 is the causal pathway considering the drug market as a key contextual factor

Figure 3 shows the key contexts, mechanisms, and outcomes that are a part of our OPC programme theory, including the elements of the three more specific causal pathways which we highlight within this theory. The general structure of the programme theory is based on the critical realist assumption that the components of an intervention can, in specified contexts, trigger underlying causal mechanisms. In this case, the relevant OPC intervention components in our retroductive analysis are: the provision of space to consume illicit drugs that is safe and hygienic, including spaces to inject and/or inhale these drugs; providing personal care at the OPC including practical support; care for injection-related wounds and cutaneous infections; educating service users about safer ways to consume (e.g. injecting practices); providing refuge from violence, which may be gender-based and/or related to the drug market; emergency response in instances of drug overdose, which can include administering oxygen and naloxone; other drug-related services (e.g. detoxification, treatment for substance use disorders) that can be provided on-site or by referral to other services.

There are some general contexts which may affect the outcomes of these interventions across more specific contexts. These general contexts include how supportive the political and legal climate is of OPC interventions. More restrictive climates restrict the availability of OPCs and the range of services they provide. In some countries—like the UK, USA, and Mexico—national and local government stands in the way of agencies opening OPCs. Even in countries that have been more supportive of harm reduction services—like some parts of Canada—governments place limits on who can use OPCs (e.g. by age or pregnancy), on the practices that can happen within them (e.g. banning or allowing peer-to-peer assisted injection) [38–40], and support can be withdrawn with political change [41]. In Germany and the Netherlands, there are several OPCs which provide space for drug use by inhalation [42]. This would be illegal in an OPC in the UK, where the Misuse of Drugs Act 1971 would criminalise people who ran such a space [17, 43].

The political climate also influences policing strategy, which influences the use and operation of OPCs. People who use drugs in some places report using OPCs as places of refuge from police surveillance that force them to hide and rush their street-based drug use [44]. There is a different relationship with the police in places, like Copenhagen, which have adopted a mode of policing which is more oriented towards harm reduction, with an ‘area of non-enforcement’ around the largest OPC in the city [45]. The political climate for the development of the non-OPCs and enforcement zone in Copenhagen was set by the election of a centre-left government which aspired to ‘put an end to marginalization, exclusion and unworthy living conditions’ [46].

The type of drugs that are used in the local market has a major impact on the operation and outcomes of OPCs. Places where the drug market is characterised by both inhalation and injection of substances like methamphetamine, cocaine, and powerful synthetic opioids (such as fentanyl) have different needs to those which mostly see injecting use of heroin. So far, this has affected the operation of OPCs in North America far more than those in Europe, as there has been less presence of potent synthetic opioids in European drug markets [47]. But there are worrying signs of increasing presence of potent synthetic opioids in European drugs markets [29, 48].

The presence or absence of peer workers at the OPC is commonly reported, in qualitative studies, to make a different to their operation and effects. People who have their own experience of street-based drug use can have an important role as peer workers in building trust between the OPC and service users [49–51]. Employment of peer workers at OPCs is more commonly reported in Canada and Australia than in Europe, although users and potential users of OPCs have expressed a preference to work with—in the words of one English research participant—‘staff that are users or ex-users because they know and care. Not someone that is just there as a job and for the money’ [52]. We found no studies that demonstrated that the engagement of peer workers improves measurable outcomes, but multiple qualitative studies—and a systematic review by Mercer et al.—note the importance of peer workers in creating an environment of safety, trust and inclusion for people who use drugs in OPCs [20, 32, 49–51, 53–59].

In reviewed documents there were a wide range of outcomes attributed to OPCs. The main reason for setting up OPCs was to save the lives of people who are involved in street-based drug use. Early studies tended to focus on the effects of OPCs in reducing HIV transmission [4, 5]. More recent research tends to focus on OPCs as a response to toxic drug poisoning deaths [60]. But the effects of go beyond preventing infections and reversing overdose. Our retroductive analysis identified a range of other important outcomes observed from OPCs. These include reductions in risk behaviours that can lead to the transmission of blood-borne viruses and to injection-related wounds and cutaneous infections [61–67]. Engagement with OPCs can also lead to general improvements in the wellbeing of service users through more general improvements in health, reduced exposure to violence, more stable housing and achievement of control over drug use through treatment for substance use disorders [68–71].

Appendix 1 shows a list of contexts, mechanisms, and outcomes that we coded. Below, we show how the general, revised programme theory presented in Fig. 3 works in more detail by applying it to specific contexts using the three key CMO configurations that we retroductively identified for these contexts.

1. Particularly for people who are unstably housed or homeless, OPCs provide a safe and supportive place for drug consumption where they can also access care, creating the outcome of a reduction in risk behaviour and improvement in overall health and wellbeing. This can be affected by the dynamic contexts of type of drug, modality of use, and time spent at the OPC.

OPCs can provide a non-stigmatising space for people who face multiple forms of marginalisation [68, 72–74], particularly for people who are unstably housed or homeless. By unstably housed, we mean people who are living in temporary accommodation in which they have no rights of tenure and ‘no private and safe space for social relations’ [75], such as shelters and single-room occupant housing. People who use drugs who are homeless or unstably housed are more likely to report willingness to use a potential OPC, and actual OPC users are indeed more likely to be homeless or unstably housed [5, 67, 76–79]. Conversely, people who use drugs who are stably housed may prefer to use at home even if an OPC is available [80–82] and the most common reason to cease using an OPC seems to be the availability of a safe private space [83]. In The Netherlands, OPC hours were cut following a reduction in need due to increased availability of social housing [84]. The Dutch government has invested in social housing following the ‘housing first’ principle, reasoning that providing housing results in the prevention of long-term care needs, crime, and street nuisance [85].

Homelessness and unstable housing are also related to higher rates of public drug use [86–88]. This is another relevant and dynamic context for OPCs. Willingness and interest for using an OPC are linked to using drugs publicly [89–91]. Initiation of OPC use is significantly associated with public injection [92]. Service users report being motivated to use an OPC as it reduces the risks related to using drugs outdoors [20]. Injecting drugs in public increases the risk of overdose due to the rushed nature of the drug consumption, often in bad-lighting conditions, out of fear of being interrupted or detected [93–95], as well as the greater likelihood of sharing used needles [88]. The availability of housing is therefore a key context. An increase in public drug use was noted in British Colombia after reductions in housing support for people with mental health problems, which coincided with the effect of the economic crisis of the last 2000s in increasing homelessness [96]. An opposite effect was observed in Amsterdam, where increased provision of housing for people who use drugs was associated with a reduction in demand for OPCs [2].

An important mechanism of OPCs in this context seems to be the feelings of safety and trust, both physically and psychologically. This underlying mechanism is produced by the intervention components of the provision of a safe, clean, and supportive space for consumption where people do not have to rush [26, 97]. This safe, non-stigmatising space also protects people who use drugs from intrusive and violent policing practice, from the stigmatising gaze of passers-by, from drug scene violence, and bans from emergency shelters, further facilitating a feeling of safety and inclusion [21, 98, 99].

Another important intervention component in this causal pathway is access to care for injection-related cutaneous infections and wounds, which are found to be more common in people experiencing homelessness or unstable housing due to the risks of public injection and a lack of access to care [100]. Another healthcare need of people who are unstably housed that can be met in OPCs is access to treatment for HCV and associated liver damage [101]. OPCs can also provide access to better personal care, including showers and washing machines [101, 102]. The risks related to the use of shared needles, or single-use equipment used multiple times, rushed injection, and a lack of hygienic preparation for injection—which are all more common in people who are homeless and inject in public places—are further mitigated by the provision of advice and education on safer injection and the provision of sterile equipment [18, 89, 91, 103, 104].

In these contexts, OPCs can cause a reduction in risk behaviours, such as street-based injection, injecting in a less rushed manner, reduced syringe sharing, and improved injecting hygiene [13, 15, 16, 105–108]. This has positive effects for injection-related injuries, morbidity, and overall health [13, 109]. As a user of INSITE told Fast et al. [104], ‘I learned how to fix myself properly in there…I think it’s had an effect on, well, I know it has had an effect on my health.’

Taken together, the literature provides evidence for the causal pathway between homelessness and public injection (contexts), feelings of safety and trust, and experiences of inclusion (mechanisms) underlying the provision of a safe, non-stigmatising, supportive space for use, access to health and personal care, advice on safe consumption and provision of sterile equipment (intervention components), and a reduction in risk behaviour and improvement in drug-use related injuries and overall health (outcomes). The interaction between the described contexts and mechanisms in this causal pathway can be further affected by the following dynamic contexts: type of drugs, modality of use (injection or inhalation), frequency of use, and time spent at the OPC. For example, people who use cocaine may prefer to use drugs privately/alone, because this drug can make them feel paranoid [103], although the higher frequency of injecting of cocaine relative to opioids may overcome such reluctance and trigger more frequent use of an OPC, as was observed at the first (unsanctioned) OPC in the UK, which operated in 2020 and 2021 [110]. Regarding time spent at an OPC, more frequent visits give OPC staff the opportunity to provide more personalised education and advice [104].

2. OPCs have the potential to work for people by offering respite from drug scene violence (especially those who identify as women, non-binary, or trans, and for marginalised ethnic groups). This can be affected by the type of drug, staff attitudes, and time spent at the OPC. OPCs may work differently for different gender identities especially for women who might require assistance to consume drugs, which many OPCs are legally unable to offer.

Gender identity seems a key pre-existing contextual factor for the use of OPCs. Across a wide variety of settings, the majority of OPC users are white cisgender men [78, 101, 111–115]. The reviewed studies suggest that OPCs act as a safe haven from police, drug scene, public violence, and harassment for men [71]. Conversely, there seem to be significant barriers for women, non-binary people, and trans people to access some OPCs [57, 116], although there is a lack of data on the experiences of non-binary and trans people. Furthermore, considering intersectionality, women who belong to racially minoritised groups experience even stronger inequalities in OPC access [117, 118]. Interestingly, female gender has been associated with willingness to use an OPC [119] and with willingness to frequently check drugs in an OPC [120], suggesting that women want to use services that can reduce the risks of their drug use.

Vulnerabilities around gender intersect with the systemic racism experienced by people from marginalised ethnic groups, including people who are racialised as Black and members of Indigenous and Aboriginal communities. Members of these groups have been reported to be over-represented in the generally disadvantaged communities that are served by OPCs [121, 122]. In early consideration of the establishment of the Insite OPC in Vancouver, it was considered possible that previous experiences of racism may deter people from using OPCs [6]. This concern seemed to be borne out, for example, in a study of an unsanctioned OPC in the USA where women and people from racialised minorities were less likely than male and white members of a street-recruited cohort of people to use the OPC [123]. However, Insite was reported to be received well by members marginalised communities. The study that suggested that Insite contributed to a greater fall in deaths in its immediate vicinity than was observed in other parts of Vancouver also found a concerning increase in deaths among women and First Nation people in areas that were not served by Insite [124]. In response to such concerns, some OPCs have paid particular attention to issues of cultural safety. For example, at Canada’s first women-only OPC—which opened in Vancouver in 2017—specific efforts were made to engage women from Indigenous communities, including the employment of Indigenous women in the peer staff group, and inclusion of Indigenous artwork and practices in the design and routines of the OPC [118].

Some other OPCs are not experienced as safe spaces by women. Due to the majority of their users being male, they can be experienced as ‘masculine space’ [117]. Some women and trans people have told OPC researchers about their ongoing experience of being hassled by men for drugs or money as they are perceived to have got them through survival sex work [125]. Kennedy et al. [13] report that many women avoid OPCs due to perceived threats of violence. Similarly, Kerman et al. [98] describe that women and trans service users feel a lack of belonging in the OPC space because of abuse and judgement.

Another context which can impede access to OPCs for women is that women, including disabled women, are more likely to need assistance when using drugs [6, 116, 117], exposing them to an increased risk of overdose, HIV infection, injection-related injuries, and the experience of violence [100, 126, 127]. The practice of assisted injection is often part of a (heterosexual) relational dynamic, sometimes characterised by violence, creating a certain type of dependency. McNeil et al. [126] report a male participant saying that.

“me and my wife, we stick together. We get high together and Insite don’t allow that. I am the only one who shoots my wife up. She can’t shoot herself and she won’t let no one else shoot her up. Actually, I won’t either, because this is our little thing. She brings in the money and I take care of keeping the drugs.”

In the early studies in Switzerland, it was observed that the man might first inject himself and then inject the woman with the same needle, so increasing risks of infections [4]. It is important to note that in addition to assisted injection occurring within—sometimes violent—relationships, it can also be part of mutual support between people who use drugs, characterised by feelings of kinship [40]. Nevertheless, assisted injection in OPCs is prohibited by law in several jurisdictions where they operate, disproportionately leading to the exclusion of women from these spaces [13]. This illustrates the potentially constraining context of the legal environment that hinders access to OPCs for some particularly vulnerable groups that could benefit from the services offered by OPCs, leaving them to use drugs in less safe environments [22, 59, 126, 128].

Xavier et al. [59] suggest that allowing assisted injection at OPCs could trigger feelings of empowerment and autonomy (mechanisms) for women, making them less dependent on controlling and abusive relationships, or on injection in unsafe environments. However, McNeil et al. [126] suggested that it may exposed women to more violence and control. More recently, there have been Canadian pilots of services that allow supervised peer and nursing staff injection, in ways that supports women’s autonomy and choice in their drug use [117]. The development of trans-inclusive women-only policies, hours, or spaces have also been suggested as another solution to the continuation of gender-based intimidation and violence [59], and these have been welcomed with great appreciation where they have been made available [6, 118].

The causal pathway described here suggests that OPCs provide respite from police, drug scene, and public violence for men. They have the potential to offer women, trans people, and non-binary people—including those with intersecting ethnic identities and experiences of racism—who may be exposed to violent relational dynamics for assisted injection (contexts) a safe space for consumption where they can receive safe assisted injection if needed, and respite from violence (intervention components) triggering feelings of safety, empowerment, and autonomy (mechanisms), leading to reduced risk of overdose, drug scene violence and injection-related injuries (outcomes). However, the pre-existing legal and political context may not allow assisted injection and men may continue to exercise gender-based violence and intimidation in ways that reduce women’s access to OPCs.

3. Features of the local illicit drug market and consumption patterns affect what will be the most effective type of service provision for OPCs. If service provision is aligned with the local drug market, the number of overdoses and deaths can be reduced through drug checking, effective overdose response, and advice on safe consumption.

The third causal pathway focuses on the local illicit drug market and the type of drugs it supplies. This is a particularly important learning point that comes to Europe from North America, where potent synthetic opioids came to dominate the market for illicit opioids, since the early 2010s, leading to a huge escalation in deaths [8]. This has not happened to anywhere near the same scale in Europe, but there are worrying signs of fentanyls and nitazenes entering European markets [48, 129]. The most recent data on drug-related deaths in Scotland also leads to concern over the presence of the potent tranquiliser xylazine. Toxicological tests on the first person to be reported as dying with xylazine in Europe also found traces of heroin, fentanyl, pregabalin, diazepam, methadone, and alcohol [130]. This shows the importance of poly-substance use as a dynamic contextual factor for overdose deaths.

The availability or absence of multiple types of drugs can impact the use, features, location, and service provision of OPCs. There are several aspects of the local drug market that play a role. Firstly, the dispersion of the drug market is important, due to the fact that the effects of OPCs can be limited to the immediate vicinity of the OPC [124]. In some places, such as New York City, the drug scene is dispersed across the city, which creates a need for multiple OPCs to increase chances of a significant impact [131]. Conversely, the drug scene in Vancouver is relatively concentrated in the Downtown East Side, justifying the need for OPCs located in that neighbourhood [103]. Some European countries—including Germany, Switzerland, the Netherlands, Denmark, Spain and Portugal—have responded to this need by creating multiple OPCs in the cities that are most affected [132].

In markets where synthetic opioids have contaminated or replaced the supply of heroin, OPCs can reduce the associated increase in overdose death risk [111, 117]. The higher potency of synthetic opioids like fentanyl and their bio-availability when inhaled has changed modes of use in several drug markets in North America, creating an increased need for OPCs to provide spaces for use by inhalation. This has long been the practice of OPCs in the Netherlands, where use of heroin by injection was largely replaced by inhalation in the 1990s, when a campaign was run to encourage people to move to ‘chasing the dragon’ on the high-quality foil that was provided to them [133]. Barcelona’s location on the Mediterranean coast provided easy access to white heroin, which is easy to mix with water for injection [134], whereas the drug supply and culture of the Netherlands has fostered lower levels of heroin use by injection than inhalation [135]. Inhalation spaces may not be provided due to local or national policies, space, or health and safety requirements to protect staff or other clients (for example, high levels of ventilation).

These contexts influence the effectiveness of some of the intervention components of an OPC. In places where injection drug use is high, OPCs might work best by providing access to injection spaces and sterile injection equipment. In places where the inhalation of synthetic opioids and coca products (such as crack cocaine and basuco) is high, OPCs might work best if they include inhalation facilities and sterile smoking equipment [136]. In 2004, Zobel and Dubois-Arber reported that OPCs in four Swiss cities had recently opened spaces foe use of drugs by inhalation [4]. Such provision has taken longer to spread to some other countries, including Canada, because of legal barriers, such as a ban on providing equipment for safer smoking [39]. Such bans exclude people from the potential benefits of providing inhalation spaces, which were reported by 2020 to be available in at least six countries, with the largest number in Germany [42].

Furthermore, the types of drugs people commonly consume in an area and the extent to which people use multiple drugs simultaneously (polydrug use) are dynamic contexts that can affect the overdose response offered by OPC staff. For example, use of (higher doses of) naloxone and/or oxygen may be needed in areas where potent synthetic opioids are prevalent [137]. Naloxone reverses opioid overdose, but will not be effective in cases where the overdose is caused by other substances. Polydrug use will also influence what symptoms staff are looking for to precipitate an overdose response [111, 138]. Tailoring these intervention components to local needs can trigger feelings of safety and engagement through service adaption, but this will require adaptation of emergency health responses.

If the service offer of the OPC aligns with the needs related to the local drug scene, this should lead to a reduction in number of overdoses experienced and in rates of mortality [15, 83, 139], as well as a reduction in risk behaviour and improved overall health [12, 13]. Conversely, gaps in service provision that do not align with the drugs people are using, might increase overdose risk [41]. In summary, the causal pathway presented here suggests that the local drug market and legal/political environment (contexts) impact on how OPCs work, and for whom. The provided services, such as inhalation spaces, injection spaces, and overdose response (intervention components) with underlying mechanisms of feelings of safety and engagement, work better if aligned with the local drug scene, causing reductions in overdose and mortality (outcomes).

### Unintended pathways and outcomes

Bonell et al. [140] describe the importance of ‘dark logic’ models in understanding the potential harmful or unintended outcomes of public health interventions, and their underlying causal mechanisms. This knowledge can help improve interventions for the future and ensure that potential harmful outcomes are avoided.

We found a few examples of OPC services that did not produce the desired outcomes, leading to premature closure or replacement. An example is the unsanctioned OPC (tolerance room) in Sydney, Australia, that predated the sanctioned Sydney medically supervised injecting centre (MSIC). The tolerance room was established in a church as an act of civil disobedience when the government was resisting a sanctioned OPC [141]. Due to a lack of resources, the tolerance room was not able to address the most pressing local concerns surrounding drug use, and served more as a symbol of civil disobedience. This example demonstrates the importance of funding and political support (contexts) for the success of an OPC. Several other studies have noted the need for stable funding and contextual support to allow OPCs to run well [20, 20, 87, 110, 142].

A second example involves the activists and researchers in the Bronx, New York City who in 2016 set up an unsanctioned OPC in two portable toilets, aptly called ‘The Portapotty Experiment’ [143]. Although people who injected in the neighbourhood were interested in the sterile syringes the team were providing, virtually no one visited the portable toilets to use drugs. In their paper, the research team openly reflect on why their experiment did not work: ‘The portapotties taught us a valuable lesson early that day; that is, what you think is a solution to a problem might not always be seen that way by the people you are trying to help. It is always better to ask first’*.* This example illustrates the importance of consultation and collaboration with people with lived experience. If interventions are imposed from the top down without consultation, important social, contextual information can easily be missed. In this case, the context of the drug culture of sharing, fears around arrest, and acceptability concerns were missed, so the potentially helpful mechanisms of the OPC were not triggered. The authors reflect that rather than expecting potential service users to come to them, they had to go into the ‘Lion’s Den’ (the nearest open drug scene) to meet the potential service users where they were [143]. Others have also emphasised the importance of consultation with the target service user group to ensure OPCs address their needs [54, 144, 145].

A third example was set in the city of Lethbridge in Alberta, Canada. Here, a larger, medically supervised OPC was replaced with a smaller, mobile OPC. The non-profit-run medically supervised OPC was closed only two and a half years after opening, due to the resistance of a new conservative government [41]. The medically supervised OPC had an important function in the region with approximately 14,000 monthly visits, and included inhalation facilities. As a direct replacement, the mobile OPC was opened by the Provincial Health Authorities. It was seen as temporary and as requiring fewer legal permissions to be run. The mobile OPC did not include an inhalation space and was in an area with frequent police patrolling. Many service users who had frequently visited the medically supervised OPC rarely visited the mobile OPC, and the closure resulted in a perceived increase in overdose deaths and drug litter [41]. This example again illustrates how changes in the political and/or policing context can negatively impact how OPCs work and for whom.

The reviewed documents also describe a few examples of unintended outcomes of otherwise successfully operating OPCs. A notable unintended outcome is staff stress and burnout. It is not just peer workers who are emotionally and psychologically affected by the high levels of mortality, morbidity and victimisation and other needs amongst the people who use OPCs. In some studies, OPC staff report high levels of emotional and physical stress, burnout symptoms, and sometimes traumatic stress [146]. The mechanisms underlying stress and burnout among staff seem to include the emotional burden of the work, overcrowding of the facility, a lack of psychological support, and minimal financial compensation [91, 145–149]. A participant in the study by Olding et al. [149] says, ‘it’s just a weariness that you can’t even explain to anyone who doesn’t do this job’. These mechanisms seem to be particularly relevant for workers and volunteers with lived experience of problematic and street-based drug use. Compared to other professionals, peer workers reportedly experience a greater lack of support, as well as higher levels of stress in their role [51, 149]. As a coping mechanism, peer workers may increase their own drug use, which can further exacerbate stress levels [149].

Another suggested unintended outcome of OPCs is the attraction of people who use drugs to the area; the so-called ‘honey pot’ effect. There are a few articles reporting increases in public nuisance after the establishment of an OPC, including drug litter and aggressive and erratic behaviour by people leaving the OPC [150–152]. Similarly, there have been reports from conservative politicians in opposition to OPCs due to concerns about increased levels of crime [153]. However, these reports seem to be concerns without strong evidence to substantiate these claims [41, 154, 155]. The overwhelming majority of studies report stable or reduced levels of crime, no significant increase of people who use drugs in the area, and an overall improved in public amenity experience [4, 15, 68, 136, 154, 156, 157].^3^

## Discussion

### Summary of findings

In order to draw lessons for the future from the first four decades of the operation of OPCs, this article reports the first realist review of the literature on OPCs, including 391 documents to identify contexts, mechanisms, and outcomes of OPCs. We presented three causal pathways from the examined literature.

The first pathway focused on OPCs as non-stigmatising spaces, and suggested that OPCs are important physical spaces for people who are unstably housed or homeless, and as a result often consume drugs in public places. In this context, OPCs trigger a sense of safety, trust and accessibility, which can lead to a reduction in risk behaviour and improvement in drug-use related injuries and overall health [13, 89, 126].

The second pathway revolved around the context of gender identity and suggests that OPCs are prominently attended by white cis-men and offer a respite from police, drug scene, and public violence for this group [71]. However, women, trans people, and non-binary people may be especially vulnerable to violent relational dynamics. OPCs have the potential to offer them a safe space for consumption, where they can receive safe assisted injection (if they wish), injection education, and respite from violence. This may trigger feelings of safety, autonomy, and empowerment, which can lead to a reduced risk of overdose, drug scene violence and injection-related injuries. However, in practice, the pre-existing legal and political contexts often do not allow assisted injection in OPCs and women, trans, and non-binary people continue to experience gender-based violence [59, 116, 125].

The third pathway considered the impact of the drug markets in the location where the OPC is situated. The services provided at an OPC should align with the needs of those who use drugs and the specific substance, and through the mechanism of safety and engagement can lead to a reduction in overdoses and drug-related mortality. However, the local political context can form a constraining pre-existing contextual factor for the extent to which certain services can be provided [15, 41, 160].

We also discussed potential unintended outcomes of OPCs and the preceding underlying mechanisms in ‘dark logic’ models, which have rarely been considered by previous reviews of the literature on OPCs, perhaps partly due to the relative lack of attention to unintended consequences other than crime and community concerns in the primary studies [140]. We described three prominent examples from the literature of OPCs that closed prematurely due to unintended outcomes as a result of interactions with certain non-conducive contexts (e.g. political environment, financial resources, lack of consultation with people who use drugs). These examples provide essential information about the contextual conditions required for OPCs to work.

### Comparison with existing literature

Similar to earlier reviews, we identified that OPCs can reduce the number of overdoses, reduce injection-related risk behaviour, reduce mortality rates, create a sense of inclusion for people who use drugs, reduce public drug use, and provide respite from drug scene violence and stress [13, 15–17, 21, 23, 157]. This article offers novel knowledge on *how* and *why* OPCs work, not just ‘if’ they work on outcomes of interest. It does so in more detail and with more specific attention to the need to inform future developments than our previous presentation of the main causal pathway [26]. Here, we provide more information on the fuller programme theory, including the causal paths of unintended outcomes.

Despite not applying realist analysis, some previous reviews do allude to mechanisms and contexts that were identified in the current study. For example, Potier et al. [16] mention the importance of the promotion of safe injection conditions, aligning with the mechanism of the experience of safety. McNeil and Small [21] describe the limiting effects of the legal and policy context, which was confirmed in the current study. This review complements the previous research literature by systematically analysing context, mechanisms, and outcomes, and describing three more specific causal pathways. Through the exploration of the first ‘dark logic’ model of OPCs, it suggests what to avoid in future design and delivery of OPCs.

Some other reviews in this area have concluded that we need more research, using more rigorous methods, before we can know whether it is worth investing in the establishment of OPCs [35, 36]. We would welcome such research, but do not think that we need to wait for it to be carried out before using the lessons that we can take from the four decades of experience with OPCs that we have reviewed here. As supply of opium from Afghanistan runs out, following the Taliban’s 2022 ban, we are seeing concerning signs of increased prevalence of synthetic opioids that are even more potent than fentanyl [29, 48]. OPCs may have an important role to play in a comprehensive response to such threats [161].

### Limitations and reflections

There are some limitations to the present study. Firstly, we only included documents that were published in English; several countries hosting OPCs do not have English as their first language, however, many have published evidence in English. Although these spanned a large variety of different countries, it is possible that we may have missed important information written in other languages. Secondly, other review types, such as systematic reviews may weight findings on the quality of evidence such as risk of bias; the diversity of methods used in the papers offering rich, relevant, and rigorous information as recommended by Dada et al. [31]makes this challenging. The current study did not exclude based on research method, which could be viewed as a limitation. As explained above and in the literature on realist methods, realist research asks *why and how* an intervention works, as opposed to seeking a definitive answer on *if* it works [162]. Realist theory assumes that interventions do not follow universally generalisable laws but instead depend on specific, contingent configurations of contexts and mechanisms [163]. So realist reviews do not exclude documents based on methodology. By drawing on research that uses a wide variety of methods, we have been able to provide a fuller picture of both the intended and unintended mechanisms and outcomes of OPCs.

Lastly, we reflect on the limitation of our subjective viewpoints and its influence on interpretation and theory development. Realist analysis cannot be independent, instead making clear the decisions taken to allow readers to appraise. There are however strategies to mitigate the risks of bias. For example, we made use of realist triangulation to allow for the convergence of multiple perspectives substantiating our findings and protecting objectivity. We have used multiple literature sources, multiple researchers conducted the analysis who draw on different perspectives e.g. health services (ZK), psychology (JRK, GWS), sociology (SSB), public health (GWS), and criminology (AS). In addition, multiple research designs were included, and multiple stakeholders (including those with living experience) were consulted for the initial and final programme theory to maximise the generalisability and reliability in so far as is possible.

## Conclusion

From the research on OPCs that have operated over the last four decades, we can conclude that OPCs can work well for certain populations, including white cis-men, people who are homeless or unstably housed, and those who are involved in street-based drug use. OPCs have the potential to work better in future for women, trans people, and non-binary people under certain conditions including accounting for injecting support, legislative change for assisted injecting, advocacy, and (trans-inclusive) women-only hours or facilities. In addition, there is potential for OPCs to work well for people for whom inhalation is the modality of drug use if the legal and resource context allows OPCs to provide inhalation facilities. The most important, common mechanisms of current OPCs across all three causal pathways include the experience of safety through the provision of a safe place for people who use drugs, safe consumption education and advice, protection from violence, and timely and adequate overdose response. There are several pre-existing and dynamic contextual factors that should be considered for OPCs to work well in future; in particular: the political and legal context, the type of drugs used in local drug markets, and the modality of use and the importance of consulting with potential and actual users of the service, as well as other people who live and work nearby.

This realist review provides useful information for policy makers, practitioners and researchers on how to implement and evaluate OPCs to maximise their future benefits. This is an important task in the context of the ongoing public health crises of drug-related deaths in the UK and North America, and the possibility of increasing deaths from synthetic opioids worldwide. By using the knowledge we have synthesised on what works, for whom, in what context, and what does not work, future OPCs can be optimised to reach the most marginalised groups, save their lives, and empower them in making informed health choices.

## Supplementary Information

Supplementary material 1.

## Data Availability

No datasets were generated or analysed during the current study.

## Abbreviations

CMO: Context-mechanism-outcome
CP: Causal pathway
MSIC: Medically supervised injecting centre
OPC: Overdose prevention centre
RAMESES: Realist and MEta-narrative evidence syntheses

## Footnotes

1 In various places and forms, OPCs are also known as drug consumption rooms, supervised consumption sites, safer injecting facilities, overdose prevention sites, and various other terms [2].

2 We use the term ‘illicit’ to refer to the use of controlled substances other than by medical prescription. This may include use of medicines by people who do not have a medical prescription for them, or use of substances obtained through illegal supply.

3 Since we completed our search, there have been two new studies of crime trends following the opening of two OPCs in New York City. One found no impact on crime levels [158]. The other found an impact on levels of property crime in the vicinity of one of these OPCs, but not the other [159]. Hall and Ratcliffe [159] suggest that the difference between the two sites can be explained by the local contexts; i.e. a large shop had recently opened near one of the OPCs, and this provided ample opportunities for shoplifting by people who were using the OPC. This again shows the importance of local contexts in influencing the outcomes of OPCs.
