# Supplementary material for: Four decades of overdose prevention centres: lessons for the future from a realist review
Source: Harm Reduct J. 2025 Mar 20;22:36. doi: 10.1186/s12954-025-01178-z (PMC11924616; doi:10.1186/s12954-025-01178-z)
Supplement: Supplementary file 1 — Supplementary material 1. [file 12954_2025_1178_MOESM1_ESM.docx]

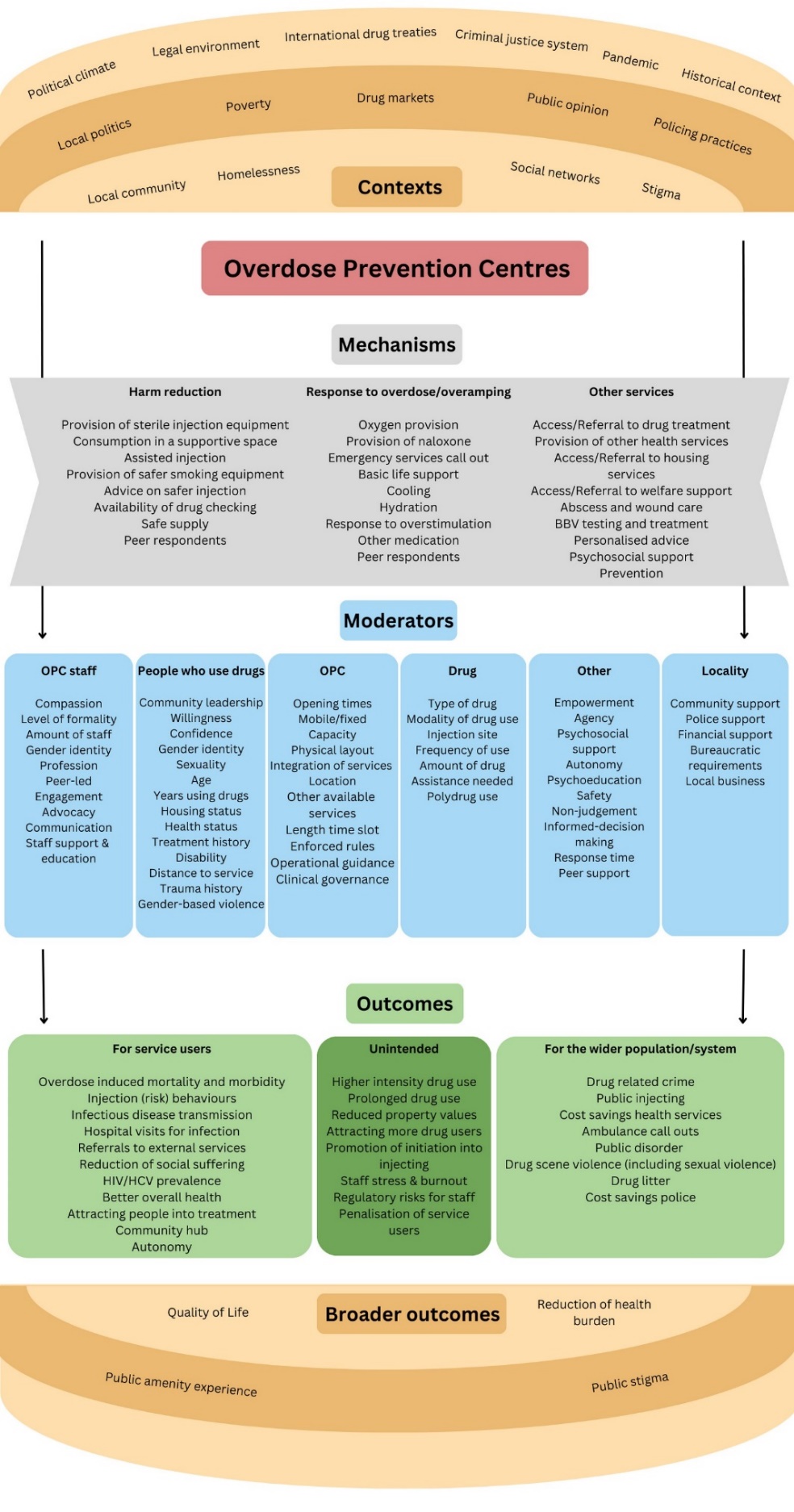
***Supplementary material:*** *Appendix 1: A diagram of the initial rough programme theory, showing posited contexts, mechanisms, and outcomes of OPCs.*
